# Locoregional Therapies for Hepatocellular Carcinoma with Portal Vein Tumor Thrombosis

**DOI:** 10.3390/cancers13215430

**Published:** 2021-10-29

**Authors:** Kylie E. Zane, Mina S. Makary

**Affiliations:** Department of Radiology, Ohio State University Wexner Medical Center, Columbus, OH 43210, USA; kylie.zane@osumc.edu

**Keywords:** HCC, portal vein tumor thrombus, TACE, SIRT, ablation, TARE, Y90, locoregional therapy

## Abstract

**Simple Summary:**

Liver cancer is a leading cause of cancer deaths in the United States and is frequently complicated by a condition called portal vein tumor thrombus, which indicates advanced disease with a short life expectancy. Recent advances in treatment of portal vein tumor thrombus have been shown to improve life expectancy in multiple trials and the evidence supporting these treatments is reviewed here.

**Abstract:**

Hepatocellular carcinoma is the fourth leading cause of cancer worldwide, and the fastest increasing cause of cancer mortality in the United States. Its propensity for vascular invasion leads to the presence of portal vein tumor thrombus in up to half of patients. PVTT results in a classification of advanced disease, given the risk recurrence secondary to intravascular spread, and formal guidelines recommend systemic therapy in these patients. However, recent advances in locoregional therapies including TACE, TARE, and ablation have demonstrated the potential to drastically improve overall survival in patients with HCC complicated by PVTT.

## 1. Introduction

### 1.1. Hepatocellular Carcinoma

Hepatocellular cancer is the fourth leading cause of cancer deaths. The incidence and mortality are highest in East Asia and Africa, but HCC is the fastest increasing cause of cancer mortality in the US [1]. The strongest risk factor for HCC is cirrhosis, present in 80–90% of HCC cases, and HCC is the leading cause of death in cirrhotic patients. Most commonly, HCC develops from cirrhosis due to hepatitis B (HBV) or C infection, alcohol consumption, or diabetes or obesity-related NASH. The etiology of HCC has considerable geographic variation: in Asia and Africa, HCC secondary to HBV accounts for roughly 60% of cases, but only 20% in the United States and Europe [1]. Given that chronic hepatitis B and cirrhosis are well-established risk factors for HCC, screening ultrasound with or without AFP is recommended every 6 months in these groups and has been associated with a 37% decrease in HCC mortality [2]. Definitive diagnosis is made with a triple phase CT demonstrating the presence of lesions with arterial enhancement and delayed washout, a finding that has a sensitivity of 89% and a specificity of 96% for HCC [1].

**Figure 1:**
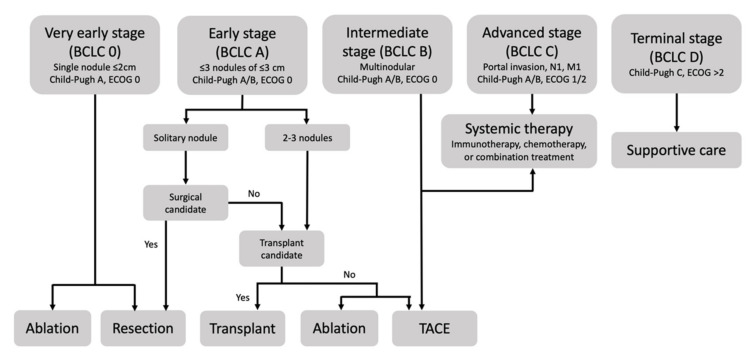
The Barcelona Clinic Liver Classification defines five stages of hepatocellular carcinoma based on assessment of disease distribution, liver function, and performance status and recommends therapy based on stage.

Despite screening recommendations for patients with cirrhosis or hepatitis B, many patients are diagnosed with advanced disease after developing abdominal pain or weight loss. Prognosis and therapy depend on tumor stage, which is defined by the Barcelona Clinic Liver Cancer (BCLC) in the United State and Europe. This staging system combines three factors: disease extension, liver function status as defined by the Child–Pugh score, and Eastern Cooperative Oncology Group (ECOG) performance status (Figure 1). Very early stage HCC, or BCLC 0, is defined as a single nodule less than 2 cm, a Child–Pugh score of A and an ECOG score of 0. Early stage HCC, or BCLC A, is defined as one to three nodules less than 3 cm, a Child–Pugh score of A or B, and an ECOG score of 0. Intermediate stage HCC, or BCLC B, is defined as multinodular HCC, and the functional and performance criteria are unchanged. Advanced stage HCC, or BCLC C, is defined as the presence of portal invasion, regional nodal metastasis, or distant metastasis, a Child–Pugh of A or B, and an ECOG of 1–2. Finally, terminal stage HCC, or BCLC D, is defined as Child–Pugh C or ECOG > 2 [3].

According to the BCLC, early stages are treated with resection, ablation, or transplant, intermediate stages are treated with transarterial chemoembolization, and advanced stages are treated with systemic chemotherapy. For early, intermediate, and advanced stages, survival is approximately 36, 16, and 6 months, respectively, in patients with preserved liver function (Child–Pugh A) [1]. However, recent advances in locoregional therapies including transarterial chemoembolization (TACE), transarterial radioembolization (TARE), ablation, radiotherapy, and hepatic intra-arterial infusion chemotherapy (HAIC) have demonstrated the potential to improve overall survival beyond six months in patients with advanced HCC complicated by PVTT.

### 1.2. Portal Vein Thrombosis

The liver receives a dual blood supply from the portal vein and the hepatic arteries. The portal vein is the primary blood supply to the liver, providing an estimated two-thirds of blood flow in the normal case. In a healthy, non-cirrhotic liver, the potential ischemic effects of portal vein thrombosis are mitigated by two compensatory mechanisms: dilation of the hepatic arteries and the rapid development in a matter of days of a system of venous collaterals adjacent to the portal vein, termed cavernous transformation. To maintain perfusion through this collateral system, portal pressures increase, which increases the risk of developing varices and associated bleeding [4]. In cirrhotic patients, the pathophysiology of portal vein thrombosis is related to the increased resistance to flow in the hepatic parenchyma secondary to fibrosis. This in turn slows portal flow rates, increasing the propensity for thrombus formation [5]. Additionally, like many cancers, HCC promotes a hypercoagulable state and also has a propensity for direct vascular invasion of the portal vein. The presentation of portal vein thrombosis is variable and may include abdominal pain, nausea, vomiting, and diarrhea in partial occlusion. In complete occlusion, it may present with abdominal pain and associated decompensation of chronic liver disease including ascites and variceal bleeding. Diagnosis of portal vein thrombosis is made with either Doppler ultrasound or contrast-enhanced CT, and can be can be classified as acute, subacute, or chronic. Treatment with anticoagulation is first line, and intervention can be considered in refractory symptomatic patients or those with complications from variceal bleeding.

HCC has a propensity for vascular invasion, particularly of the portal vein, resulting in a specific case of portal vein occlusion termed portal vein tumor thrombosis (PVTT), which does not respond to anticoagulation. PVTT affects between 35–50% of patients with HCC [6] and the presence of PVTT designates advanced disease (BCLC stage C). The poor prognosis is due to the risk of intravascular metastatic spread, increased risk of complications such as bleeding esophageal varices [7], and impaired liver function due to occlusion of the liver’s primary blood supply and worsening portal hypertension. For patients with PVTT, transplant is contraindicated, and the National Cancer Comprehensive Network (NCCN) recommendation for advanced HCC is locoregional therapy or systemic treatment. First line systemic treatment is with atezolizumab plus bevacizumab [8]. While sorafenib has long been the standard of care for these patients based on the results of two randomized controlled trials [9,10], combination atezolizumab plus bevacizumab recently demonstrated superiority in a landmark trial [11]. In this trial, combination therapy was associated with a hazard ratio for death of 0.58 compared to sorafenib (*p* < 0.001), improved overall survival at 12 months (67.2% vs. 54.6%), and improved progression-free survival (6.8 vs. 4.3 months). The NCCN guidelines acknowledge that there is currently insufficient evidence to recommend systemic therapy over locoregional therapy [8], and recent studies of locoregional therapies such as transarterial chemoembolization (TACE), transarterial radioembolization (TARE) and ablative therapies have demonstrated benefit in HCC with PVTT in select cases.

### 1.3. Portal Vein Tumor Thrombus Classification

The portal vein is formed from the confluence of the superior mesenteric vein and the splenic vein, and within the liver, it divides into a left and right branch. Various further subdivisions supply the hepatic sinusoids. Thus, portal vein thrombus may involve distal branches of the portal vein and affect only a segment or a lobe, or it may involve the main trunk of the portal vein, affecting the entire liver.

**Figure 2:**
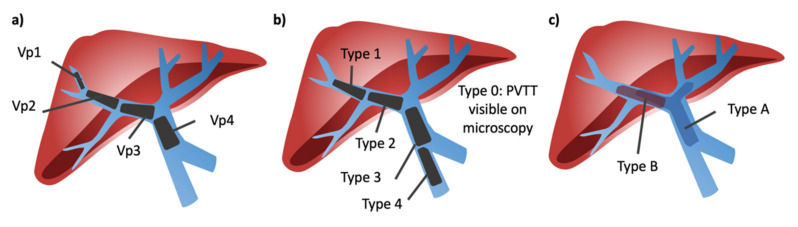
Portal vein tumor thrombosis classification systems. (**a**) Liver Cancer Study Group of Japan classification; Vp1 = thrombus located beyond second order branches, Vp2 = thrombus located in the second order branches, Vp3 = thrombus located in the first order branches, Vp4 = thrombus located in the main portal vein. (**b**) Cheng classification; Type 0 = PVTT seen only with microscopy, Type 1 = PVTT in the second-order segmental branches, Type 2 = PVTT in the right or left portal vein, Type 3 = PVTT in the main portal vein, and Type 4 = PVTT in the superior mesenteric vein. (**c**) Xu classification; type A = thrombus in main portal vein or both right and left portal veins, type B = thrombus in either right or left portal vein.

In order to understand the literature regarding HCC with PVTT, it is important to be aware of the various classification systems commonly used—The Liver Cancer Study Group of Japan, the Cheng Classification, and the Xu classification—as the location of PVTT impacts prognosis and therapeutic options (Figure 2). The Vp classification system from the Liver Cancer Study Group of Japan is the most commonly used [12]. In this system, Vp0 indicates no tumor in the portal vein, Vp1 indicates tumor distal to second order branches, Vp2 indicates tumor within second order branches, Vp3 indicates tumor in first order branches, and Vp4 indicates tumor in the main portal vein. This must be carefully distinguished from the Cheng classification, where Type 0 indicates PVTT seen only with microscopy, Type 1 indicates PVTT in the second-order segmental branches, Type 2 indicates PVTT in the right or left portal vein, Type 3 indicates PVTT in the main portal vein, and Type 4 indicates PVTT in the superior mesenteric vein [13]. Finally, the Xu classification system specifies Type A as involvement of the main portal vein or both the right and left portal veins and Type B as involvement of either the right or left portal vein [14].

The need for a classification system arose in China and Japan as a result of guidelines that permit resection as a management strategy for HCC with PVTT. In literature from the United States and Europe, where these classification systems are less common, ambiguity arises when terms like segmental or sub-segmental PVTT are used to group PVTT patients. Here, segmental is used to mean any portal vein tumor thrombus outside of the main portal vein.

## 2. Locoregional Therapies

### 2.1. Patient Selection

Across studies, various criteria are used to select patients appropriate for locoregional therapy in the setting of HCC with PVTT. All studies require a Child–Pugh score of A or B (lower than or equal to 7 or 8) and ECOG performance status between 0 and 1, with the occasional inclusion of patients with ECOG of 2. Other shared criteria included minimum patient age of 18 to 20 and a maximum age of 75 to 80. Patients with extrahepatic disease or signs of hepatic decompensation, including variceal bleedings, hepatic encephalopathy, and significant ascites were typically excluded. Locoregional therapies other than TACE often required that candidates were not eligible for TACE given the preference for this treatment in BCLC guidelines. In the case of TACE, some studies required that patients were treatment naïve, as prior chemotherapy can deplete liver reserve while preserving relatively normal liver lab values. Studies were often variable on the inclusion of patients with main portal vein thrombus, though multiple studies specifically examined these populations. The extent of portal vein tumor thrombus involvement is specified where appropriate. Some studies specified thresholds for various lab parameters, for example, total bilirubin (<2–3 mg/dL, or 34.2–51 µmol/L), albumin (>2.7–2.8 mg/dL), and serum alanine aminotransferase (<5–10× upper limit of normal). Hematologic parameters, for example, thresholds for white blood cell counts to exclude active infection, hemoglobin (>8–8.5 mg/dL) and platelets (>30,000–60,000 platelets/microliter) were included in some studies. Differences in inclusion criteria between studies have the potential to influence results. For example, comparisons of locoregional trials using lower total bilirubin cut-offs correspond to longer survival times when compared to similar trials with more permissive bilirubin cut-offs [15]. Prognostic factors that are commonly associated with improved overall survival include ECOG 0, low tumor burden, and absence of cirrhosis, ascites, and extrahepatic disease [16]. Results of the following trials are summarized below in Table 1.

### 2.2. Transarterial Chemoembolization

While the hepatic arteries are not the dominant blood supply to the liver, they are the dominant supply to liver tumors (~90%) as a result of neoplastic angiogenesis. This principle forms the basis of transarterial chemoembolization (TACE) and transarterial radioembolization (TARE), which are catheter-directed locoregional therapies originally developed in the 1960s and 70s [25,26]. In TACE, chemotherapy is administered through a catheter to the hepatic arteries supplying the tumor. One method is conventional TACE (c-TACE), wherein lipiodolized chemotherapy is administered followed by embolic beads. The second method is to use drug-eluting beads (DEB-TACE), wherein the chemotherapy and the embolic beads are combined, allowing for sustained release and improved standardization of chemotherapy dosing [27]. While no differences in overall survival have been demonstrated between these two therapies, DEB-TACE is associated with fewer adverse events [28]. The most common side effect of these procedures is postembolization syndrome (PES), which presents as self-limiting right upper quadrant pain, nausea, fever, and elevated liver function tests. PES is attributed to tumor necrosis and tissue ischemia and full recovery within seven to ten days is typical. Complications include hepatic decompensation, renal injury, biliary injury, infection, and non-target embolization.

TACE has been the standard of care for intermediate disease (BCLC B) for the past two decades, after the results of two randomized controlled trials demonstrated an increased overall survival for patients treated with c-TACE (one trial used doxorubicin and one used cisplatin) compared to embolization alone [29,30]. Currently, the most common chemotherapeutic agent used for TACE is doxorubicin; mitomycin C and cisplatin are also used.

In the setting of HCC with PVTT, the NCCN and BCLC guidelines recommend against TACE due to concerns that arterial embolization in the setting of pre-existing occlusion of the liver’s primary blood supply will result in severe ischemia and compromise remaining liver function. However, the likelihood of this depends on multiple factors, including the degree of cavernous transformation, the location of portal vein thrombus, and the location of the tumor. As a result, multiple retrospective studies have demonstrated the safety of TACE in the presence of both segmental and main PVTT [31,32,33]. Regarding the choice of cTACE vs. DEB-TACE in the setting of PVTT, retrospective comparison has not revealed significantly different overall survival [32].

The survival data for TACE in the setting of HCC with PVTT is limited, comprising a meta-analysis showing improved overall survival in TACE vs. conservative management [34] and a prospective trial showing improved overall survival in TACE vs. conservative treatment in patients with both segmental and main PVTT (12- and 24-month survival rates of 30.9% and 9.2% vs. 3.8% and 0%). Downstaging to partial resection or ablation was achieved in 9 patients (10.7%) [17]. The weakness of both these studies is the lack of comparison to the standard of care, systemic therapy. In comparisons of TACE plus sorafenib vs. TACE alone in patients with segmental PVTT, retrospective studies and meta-analyses demonstrate improved survival [18,35]. The strongest evidence to date for TACE in the setting of HCC with PVTT comes from a recent RCT that demonstrated increased overall survival using TACE plus external beam radiation therapy (ERBT) compared to sorafenib (55 weeks vs. 43 weeks). In this study, 5 patients (11.1%) in the treatment arm were downstaged to curative resection [19]. Given the sensitivity of liver parenchyma to radiation, radiation damage was minimized by limiting ERBT administration to PVTT and contiguous tumors using 3D conformational radiotherapy, as opposed to lobar or whole organ radiation.

Recent and ongoing research in China explores the potential role for TACE in combination with other treatment modalities for HCC with PVTT. A recent retrospective study of placement of a portal vein stent seeded with a radioisotope of iodine, ^125^I, followed by TACE with sorafenib demonstrated a median overall survival of 10 months [36]. Ongoing trials are exploring combinations of TACE with epirubicin and resection (NCT04619342) and tumor recurrence rates after post-resection treatment with TACE vs. hepatic intraarterial infusion of chemotherapy (NCT03192644).

### 2.3. Transarterial Radioembolization

TARE uses 30-micron resin or glass beads that have been embedded or coated with a radioisotope of yttrium, ^90^Y [37]. Despite the name, this bead size is not large enough to cause significant embolic effects—in fact, successful treatment requires continued arterial blood flow. Once introduced, ^90^Y undergoes beta-decay causing radiation-induced damage to cellular DNA repair mechanisms and ultimately, cell death. The most common side effect of TARE is post-radiation syndrome, a set of non-specific symptoms including fatigue, nausea, anorexia, and fever that can persist for up to two weeks in 20–70% of patients [38]. A less common side effect is radioembolization-induced liver disease (REILD), defined by jaundice and ascites that persist 1–2 months after treatment without evidence of obstruction or tumor progression and is associated with the presence of cirrhosis and non-cirrhotic patients with prior exposure to systemic therapy [39]. Reported rates of REILD vary, occurring in 0–11% of patients with HCC, 0–20% of patients with metastatic disease, and dropping to 0–1% in randomized controlled trials for HCC and metastatic CRC [37].

TARE does not have a well-established role in the treatment algorithm for HCC, but some authors argue that TARE and TACE should be interchangeable in the treatment of intermediate HCC. Indeed, small-scale studies support similar outcomes for TARE in intermediate HCC [40,41,42] and recent data from a large, observational study in Europe reported a median overall survival of 16.5 months in unresectable HCC patients treated with TARE [16].

Compared to TACE, there is less concern for severe ischemia when administering TARE in the setting of PVTT, making it an appealing option for this patient population. Initial prospective studies suggested a potential survival benefit for patients with PVTT treated with TARE [40,41,42,43]. However, three RCTs that followed these promising findings failed to demonstrate superiority compared to sorafenib, even when TARE was combined with sorafenib [44,45,46]. One trial of TARE plus sorafenib vs. sorafenib is ongoing (STOP-HCC, NCT 0155649). Despite these negative findings, these three trials were combined in a meta-analysis that was adequately powered to demonstrate that TARE is non-inferior to sorafenib and offers a better safety profile [15]. This analysis and others suggest there may be a subset of patients with PVTT, specifically non-cirrhotic patients, patients with hepatitis B-associated HCC, and patients with preserved functional status, who would benefit from TARE over systemic therapy [47]. This is reflected in the most recent NCCN guidelines, which state that TARE may be appropriate in the treatment of segmental or lobar PVTT [8].

Another development in the use of TARE in HCC with PVTT is the concept of personalized dosimetry, also called boosted selective internal radiotherapy (B-SIRT). This approach delivers a higher dose of radiation to the tumor than standard dosimetry (>205 Gy vs. 120 Gy) without a significant increase in liver adverse events [48]. Building on previous work, a recent phase II trial demonstrated improved overall survival in HCC patients with PVTT treated with personalized dosimetry compared to standardized TARE (22.9 vs. 9.5 months), with downstaging to resection in up to 12.2% of patients [20,48,49]. Dose increases for TARE in HCC with PVTT have developed in tandem with increased dosing for TARE in HCC more generally; with data from the recent retrospective LEGACY study supporting the use of 400 Gy [50].

The use of TARE for HCC doubled from 2010 to 2015 [47]. With increased operator familiarity, the advent of personalized dosimetry, and patient data from randomized controlled trials to date, it may be possible to demonstrate a survival advantage for TARE over systemic therapy for patients with PVTT in the near future.

### 2.4. Ablation

Ablative strategies deliver thermal, chemical, or electrical energy to induce tumor necrosis. In HCC, the most common ablation methods include radiofrequency ablation (RFA), microwave ablation (MWA), and cryoablation. In these methods, a needle or probe is placed percutaneously into the tumor and thermal energy is used to induce tumor necrosis. RFA uses an electrode-tipped probe that emits an alternating current. It is most effective for small tumors (<3 cm) that are not adjacent to large vessels like the portal vein or hepatic hilum, as the flow within these vessels can act as a heat sink drawing off the thermal energy needed for successful ablation. MWA uses an antenna to generate an electromagnetic field that aligns nearby water molecules, producing thermal energy. In contrast to RFA, MWA achieves target temperatures faster over a larger area, produces more uniform heating zones, and is less susceptible to heat sink effects. Further, MWA can achieve a larger ablation zone, as multiple probes can be placed simultaneously. In cryoablation, hollow needles emit cold gas, which is then frozen to destroy tumor tissue. Finally, irreversible electroporation (IRE) is a new ablative technology that induces cell death using direct current to create holes in the cell membrane. The major side effect of these therapies is a self-limiting post-ablation syndrome characterized by fever, malaise, and chills that occurs in up to a third of patients in the week following embolization [51]. Major complications occur in less than 4% of patients and include bleeding, abscess, liver failure, and damage to surrounding structures [52].

In HCC, ablation is a curative treatment option for very early and early stage HCC (BCLC A) less than 3–4 cm. Multiple guidelines (European Association for the Study of the Liver, Asian Pacific Association for the Study of the Liver, American Association for the Study of Liver Diseases) recommend ablation for small single tumors and for patients with early stage HCC who are not candidates for surgery [53]. In the current guidelines, there is no role for ablation in patients with advanced disease, including those with PVTT. However, case studies demonstrate that direct ablation of PVTT using a coaxial approach is possible and can lead to high rates of portal vein recanalization [54]. Three RCTs over the last decade demonstrate a survival benefit when ablation is added to the treatment of HCC with PVTT. First, a comparison of cryotherapy plus sorafenib to sorafenib alone demonstrated improved survival in patients with PVTT and Child–Pugh A or B (OS 12.5 vs. 8.6 months) [21]. Second, a comparison of RFA plus sorafenib to sorafenib alone in patients with main PVTT and Child–Pugh A showed improved survival (1-, 3-, and 5-year survival rates of 63%, 30%, and 20% vs. a 1-year survival rate of 0%) [22,54]. Finally, a comparison of RFA, sorafenib and cTACE to sorafenib and cTACE demonstrated that the addition of ablation led to improved survival (OS 468 days vs. 219 days) [23]. An interesting prospective study of MWA following TACE for HCC with PVTT demonstrated improved overall survival when compared to a historical cohort of patients treated with TACE (13.5 vs. 9.5 months). Notably, this improvement was seen even with broad inclusion parameters that enrolled patients with extrahepatic disease, Child–Pugh Grade A and B, and segmental and main PVTT [24].

## 3. Other Approaches to HCC with PVTT

### 3.1. Radiotherapy

External beam radiotherapy (EBRT) is not commonly used in HCC, as the liver as a whole has low tolerance for radiation. However, the development of targeted EBRT approaches such as 3D conformational radiation therapy (3DCRT), intensity-modulated radiation therapy, and stereotactic body radiation (SBRT) that can be targeted to tumors while sparing normal liver parenchyma has led to attempts to treat unresectable HCC with external radiation. Further, the development of proton beam radiotherapy, which delivers radiation directly to the target site while minimizing off-target radiation along the entry and exit path allows for further precision [55]. These methods can be used to treat PVTT alone, HCC within the hepatic parenchyma, or both. Prospective trials of SBRT with protons or photons demonstrate acceptable tumor safety and evidence of tumor response in patients with Child–Pugh A [56,57]. Retrospective work has demonstrated a role for SBRT in patients with PVTT, demonstrating superior survival compared to conventionally fractionated radiotherapy [58]. Many comparisons between SBRT and local approaches like ablation are limited by their retrospective nature, but one recent RCT comparing proton beam radiotherapy to radiofrequency ablation demonstrated non-inferiority [59]. Thus, there may be a role for radiotherapy in the treatment of advanced HCC, though further studies comparing these approaches to currently accepted therapies such as ablation and TACE are needed.

### 3.2. Hepatic Intra-Arterial Infusion

Hepatic intra-arterial infusion chemotherapy (HAIC) is another treatment for advanced HCC and involves the percutaneous placement of a pump that delivers chemotherapy to the entire liver. Placement requires exploratory laparotomy, cholecystectomy, and devascularization of the distal stomach and proximal duodenum. While included in Japanese treatment guidelines for advanced HCC [60], it is less commonly performed for advanced HCC in the United States. In fact, recent phase 2 and 3 trials have failed to demonstrate an improvement in overall survival for HAIC versus sorafenib in advanced HCC. However, three recent studies suggest a role for HAIC in advanced HCC in the setting of PVTT [61,62]. The first demonstrated improved overall survival in a comparison of HAIC (cisplatin, 5-FU) versus sorafenib in HCC with PVTT (14.9 vs. 4.4 months) [63]. Another compared HAIC alone to sorafenib alone in patients with PVTT without extrahepatic disease, demonstrating improved overall survival (10.1 vs. 9.1 months), though no improvement in overall survival was seen for patients with PVTT and extrahepatic disease [61]. The strongest evidence comes from an RCT that demonstrated that sorafenib plus HAIC of oxaliplatin, 5FU, and leucovorin (FOLFOX) improved overall survival compared with sorafenib in patients with advanced HCC with portal vein invasion (13.4 vs. 7.1 months) [62].

In Korea, HAIC has also been combined with EBRT in a treatment approach termed liver-directed concurrent chemo-radiotherapy (LD-CCRT). A retrospective study of 152 patients with main trunk or first order branch PVTT who underwent LD-CCRT demonstrated an OS of 13.5 months, with downstaging to curative resection or transplant in 10.5% of patients [64]. A prospective trial of 47 patients who underwent LD-CCRT followed by systemic sorafenib demonstrated an overall survival of 13 months for patients with PVTT in the main or first order portal vein [65]. Given the risk for toxicity from EBRT, this treatment approach is best suited for patients with focal disease as opposed to multifocal or bilobar disease.

### 3.3. Resection

According to BCLC guidelines, resection is appropriate for patients with very early or early stage disease, limited to three nodules 3 cm or less in size. While these guidelines do not indicate a role for resection in HCC with PVTT, some studies suggest a role for resection in patients with segmental portal vein involvement [66]. Much of this work comes from Japan and China, where resection may be considered for HCC with PVTT. One such retrospective study of over 6000 patients compared resection to other treatments in patients with PVTT and found increased overall survival (2.9 vs. 1.1 years). Subgroup analysis revealed a lack of benefit for patients with PVTT involving the main portal vein [67]. A recent retrospective study from Germany found that survival increased by more than two years for resection versus TACE or TARE in patients with PVTT distal to second order branches (Vp1) (32.4 vs. 8.1 months) [68]. Given the high rates of recurrence after resection [1], gains in survival from resection are maximized when used in conjunction with locoregional or systemic therapies. In fact, RCTs from Asia, where PVTT is not a contraindication to resection, have demonstrated a survival benefit when resection is followed by TACE compared to TACE alone (13 vs. 9 months) [69,70]. Similarly, survival improved when resection was followed by systemic chemotherapy (5.1 vs. 2.5 months) [71].

## 4. Conclusions

Treatment options for HCC with portal vein tumor thrombus have come a long way in the last ten years. While official guidelines recommend systemic therapy for these patients, there is growing evidence that locoregional approaches can offer significant gains in survival for patients who were historically given a prognosis of 2 to 4 months, especially in patients with PVTT sparing the main portal vein. While more randomized controlled trials are needed, there is evidence to suggest that TACE is safe and effective in patients with PVTT, and further, that the addition of TACE to sorafenib has the potential to prolong survival in this patient population. Regarding TARE, it appears to be non-inferior to systemic therapies in terms of survival, and advances in personalized dosimetry will likely lead to further improvements in the near future. Ablation, too, can offer improved outcomes for HCC with PVTT when combined with sorafenib and other locoregional therapies. Developments in locoregional approaches, combined with developments in systemic therapies and surgical approaches will continue to improve survival for patients with HCC complicated by PVTT.

## Figures and Tables

**Table 1:** Locoregional therapies for HCC with PVTT.

Study	Type	Size	PVTT	Treatment	Outcomes
Luo 2011 [17]	Prospective	164	Vp1–Vp4	TACE	12- and 24-mos. OS of 30.9% and 9.2%, downstaging in 10.7%
Zhu 2014 [18]	Retrospective	91	Vp2, Vp3	TACE + sorafenib	OS 14 mos
Yoon 2018 [19]	RCT	90	Vp 2–Vp4	TACE + ERBT	OS 13.8 mos, downstaging in 11.1%
Venerito 2020 [15]	Meta-analysis	1243	Vp 2–Vp4	TARE	Non-inferiority of TACE to sorafenib
Garin 2015 [20]	RCT	41	Vp 2–Vp4	Personalized Dosimetry TARE	OS 22.9 mos, downstaging in 12.2%
Yang 2012 [21]	RCT	104	Vp 1–Vp4	Cryotherapy + sorafenib	OS 12.5 mos
Giorgio 2016 [22]	RCT	99	Vp4	RFA + sorafenib	1-, 3-, and 5-year OS: 63%, 30%, and 20%
Ding 2020 [23]	Prospective	80	Vp1–Vp3	RFA + TACE + sorafenib	OS 15.3 mos
Long 2016 [24]	Prospective	109	Vp2–Vp4	MWA after TACE	OS 13.5 mos

PVTT = portal vein tumor thrombus; RCT = randomized, controlled trial; TACE = transarterial chemoembolization; ERBT = external beam radiotherapy; TARE = transarterial radioembolization; RFA = radiofrequency embolization; MWA = microwave ablation; downstaging refers to the percentage of patients who were able to undergo resection or ablation following treatment.
